# Relationship between patient safety culture and staff motivation in Tunisian operating rooms: a cross-sectional study

**DOI:** 10.1186/s12913-025-13995-w

**Published:** 2026-08-06

**Authors:** Hela Ghali, Sonia Daafous, Yossra Romdhan, Ghofrane Hlaimi, Asma Ben Amor

**Affiliations:** 1 Department of Preventive and Community Medicine, Sahloul University Hospital, Faculty of Medicine of Sousse, University of Sousse, Sousse, Tunisia; 2 Higher School of Health Sciences and Techniques of Souss, University of Sousse, Sousse, Tunisia; 3 Family Medicine, Faculty of Medicine of Sousse, University of Sousse, Sousse, Tunisia; 4 Higher School of Health Sciences and Techniques of Sousse, Faculty of Medicine of Sousse, University of Sousse, Sousse, Tunisia

**Keywords:** Patient safety culture, Staff motivation, Operating rooms, Hospital Survey on Patient Safety Culture, Adverse events, University hospitals

## Abstract

**Background:**

In recent decades, patient safety culture has become a crucial public health concern. Studies indicate that the level of safety culture needs enhancement. This improvement requires the integration of human resources, which is contingent upon work motivation. Globally, avoidable patient harm remains a leading cause of morbidity and mortality, underscoring the urgent need for comprehensive safety interventions.

**General objective:**

To study the relationship between patient safety culture and staff motivation in the operating rooms of the two university hospitals of Sousse.

**Materials and methods:**

This descriptive, cross-sectional study was conducted in the operating rooms of two university hospitals in the Sousse region (April 15th - May 10th, 2024). Data collected were analyzed using SPSS version 26.0, employing descriptive statistics (frequencies, percentages, means, standard deviations) and inferential statistics (Pearson’s Chi-squared test or Fisher’s exact test for categorical variables). Our research employed two measurement tools: the Hospital Survey on Patient Safety Culture (HSOPSC) and a validated motivation questionnaire based on Maslow and Herzberg’s theories.

**Results:**

This study surveyed 236 operating room staff from two university hospitals, achieving an 81.94% response rate. Key findings revealed that no safety culture composite reached 75% positive response, and 44.92% of participants reported low motivation at work. Several HSOPSC composites were significantly correlated with motivation factors, including: “Overall perception of safety” and “Teamwork between departments of the institution” were linked to “Remuneration”, “Expectations and actions of hierarchical superiors regarding patient safety,” “Learning organization and continuous improvement,” and “Freedom of expression” were associated with “Job attributes», «Human resources” with “Collaboration”, “Frequency of reporting adverse events,” “Learning organization and continuous improvement,” and “Human resources” were correlated with “Achievement” .

**Conclusion:**

Fostering a motivating work environment that champions patient safety is essential for hospitals. This setting, built on open communication and staff empowerment, significantly reduces medical errors and improves patient outcomes. For administrators, investing in staff motivation and safety culture is a strategic imperative that boosts care quality and reputation.

**Supplementary Information:**

The online version contains supplementary material available at https://doi.org/10.1186/s12913-025-13995-w.

## Background

In recent decades, the fields of medicine in general, and surgery in particular, have undergone significant advancements, both scientifically and empirically. As a result, the care quality and safety in hospitals have become major public health concerns and a fundamental focus of healthcare systems [1].

This progress has been accompanied by an increase in healthcare activities and hospital admissions. In this context, adverse events can and do occur too frequently [2].

Indeed, a recent literature review (2024) highlighted the extent of adverse events (AEs) related to healthcare, both in terms of frequency and cost, as well as the serious consequences that can result [3].

A study conducted in 2022 in the Eastern Mediterranean region showed that the annual number of adverse events is approximately 4.4 million, and 18% of hospital admissions are linked to adverse events associated with care, leading to high rates of death and permanent disability [4].

Moreover, healthcare-related AEs result in significant financial losses. A study conducted in Poland in 2022 about Financial Expenses of the Healthcare System Resulting from the Occurrence of AEs showed that On average, EUR 1433 was spent to compensate for the negative consequences [5] .

In Tunisia, as in many other countries, the same problem is observed. A previous study conducted at the Sahloul University Hospital in 2016, assessed that the incidence density of AEs was 1.8 events per 100 days of hospitalization [6].

In the operating room, care often requires collaboration among several healthcare providers, performing different tasks in parallel, which implies inherent opportunities for miscommunication and an increased risk of AEs [7].

As a result, many countries have implemented national strategies to reduce the incidence of these AEs. Within this context, a study conducted in the Netherlands discussed methods for preventing the occurrence of healthcare-associated events essentially evaluation of safety behavior, improving procedures, and improving information and communication structures [8].

In Tunisia, a study conducted in 2012 among 116 physicians working in hospital departments at a University Hospital in Sousse revealed that among physicians, 54.7% deemed the level of care safety in their department to be acceptable [9]. Another cross-sectional study was conducted in 15 intensive care units belonging to the two university hospitals in the region of Sousse, using the Hospital Survey On Patient Safety Culture (HSOPSC) questionnaire showed that all of the composites had a score of less than 50% [10].

Enhancing patient safety in hospitals demands a multidimensional approach that goes beyond technical and organizational adjustments to include strategic human resource management. The success of healthcare institutions, particularly in fulfilling their public service mission, inherently relies on the performance and commitment of healthcare professionals.

**Fig. 1:**
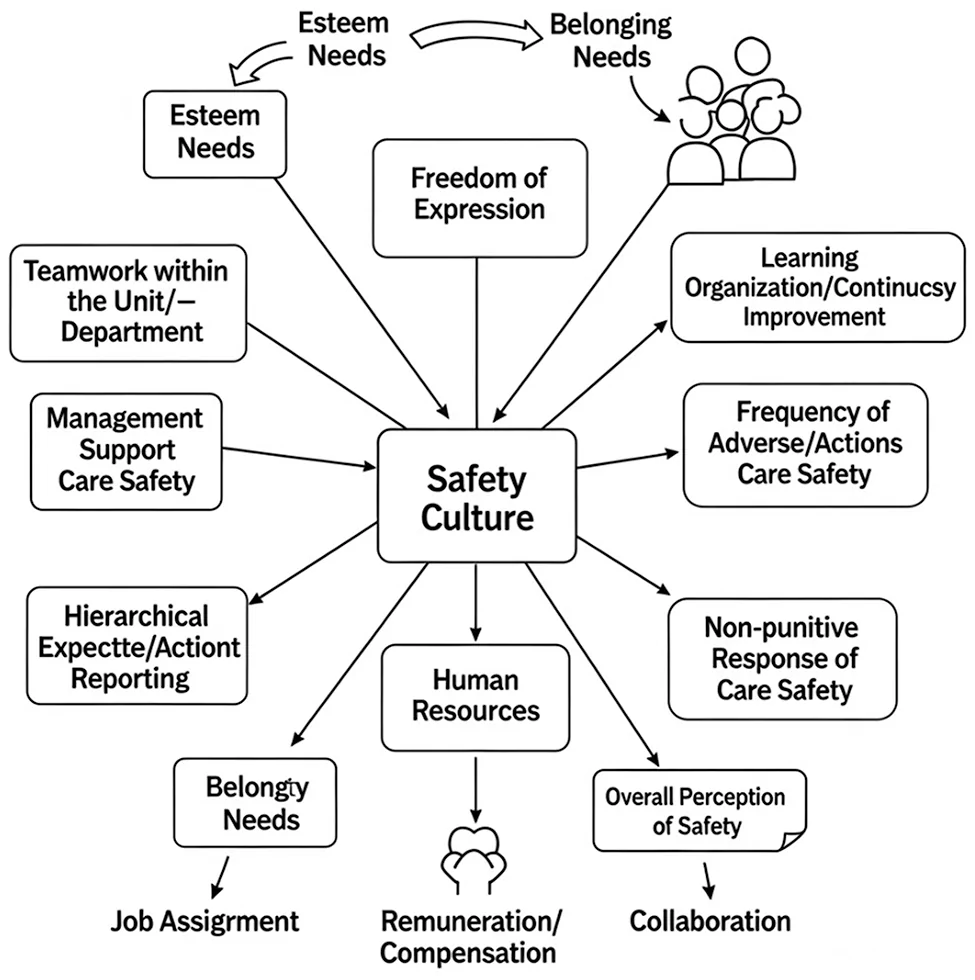
Conceptual framework: relationship between safety culture and staff motivation

Drawing on organizational behavior and motivation theories, a robust safety culture is established to foster an environment characterized by psychological safety, transparent communication, and staff empowerment. This environment directly influences caregivers’ intrinsic and extrinsic motivation (Fig. 1).

For instance, Self-Determination Theory suggests that a sense of autonomy, competence, and relatedness—often reinforced by a strong safety culture—boosts individuals’ motivation to adhere to safety protocols and actively participate in error prevention.

Indeed, the motivation of human resources is an extremely relevant element and is an essential source that contributes to improving work performance by ensuring better quality and safety of care [11].

Two studies conducted in Morocco, one at the CHU Iben Zohr in Marrakech in 2008 and the other conducted in 2012 at the CHU ALFARABI, found a motivation rate of 32.5% and 25%, respectively [12, 13].

The World Health Organization (WHO) pays particular attention to human resources for health (HRH) and the relationship between motivation and the level of safety culture in care through the adoption in 2016 of the Global Strategy on Human Resources for Health: workforce 2030 [14].

Furthermore, in 2014, research conducted at the Farhat Hached University Hospital Center in Sousse showed that more than half (65%) of the staff are “not very motivated” in their work [15].

Moreover, due to the high prevalence of demotivation among healthcare personnel, the increasing number of AEs particularly affecting operated patients, and the urgent need to measure the levels of SC in the hospital, our study is inscribed, which aims to describe the level of safety culture in healthcare among personnel working in operating rooms, evaluate the degree of work motivation among operating room personnel, assess the relationship between safety culture and staff motivation in the operating rooms of two university hospitals in the center-east of Tunisia.

## Materials and methods

### Study design

A descriptive cross-sectional study was conducted to explore our research questions and better achieve the study objectives.

### Study setting

This four-week study (April 15th - May 10th, 2024) took place in the operating rooms of Sousse’s Farhat Hached and Sahloul University Hospitals. Specific surgical specialties included neurosurgery, emergency, general surgery, orthopedics, maxillofacial surgery, burns, urology, cardiovascular surgery, Ear, Nose, and Throat (ENT), ophthalmology, and gynecology-obstetrics.

The study employed a convenience sampling strategy complemented by a voluntary response sampling approach given the nature of the survey. This method was chosen due to participant accessibility and the necessity of obtaining informed consent from each eligible operating room staff member. Population size is *N* = 288 .

### Study population

Our study included all healthcare professionals working in the operating rooms of Sousse. This population consisted of physicians, residents, senior operating room instrumentation technicians (SOIT), senior anesthesia technicians (SAT), and operating room nurses (ORN).

### Inclusion criteria

Any physician, resident, senior operating room instrumentation technicians (SOIT), senior anesthesia technicians (SAT), and operating room nurses (ORN) who agreed to participate in the study.

### Exclusion criteria

No exclusion criteria were defined, as no characteristics likely to interfere with the results were identified.

### Study instrument

Data collection took place in the two university hospitals of the Sousse region over a month, using a self-administered questionnaire after obtaining the agreement of the department heads. Questionnaires were distributed to healthcare professionals meeting the inclusion criteria and collected within a week. Healthcare professionals were contacted from Monday to Saturday and during night shifts at their respective workplaces. Brief information explaining the objectives of the work was given to the staff, requesting their participation and assuring them of confidentiality and anonymity. Two visits were made to remind participants and increase the response rate to the questionnaire. Paper copies of the questionnaire were distributed, we utilized a validated questionnaire comprising 3 sections.

### Sociodemographic data

A questionnaire was used to collect data on seven sociodemographic variables: age, gender, grade, seniority, risk management committee participation, patient safety training, institution type, and certification. The sociodemographic data questionnaire used in this study was specifically developed for this research. An English language version has been provided as a supplementary file. To validate, we ensured all questions were relevant to the research objectives and that they comprehensively covered essential sociodemographic variables.

### The hospital survey on patient safety culture (HSOPSC) [16]

A validated tool that was developed in 2004 in the United States by the Agency for Healthcare Research and Quality (AHRQ), translated into French and validated by the Coordination Committee for Clinical Evaluation and Quality in Aquitaine (CCECQA, France) [17]. Due to its good psychometric qualities, it is the most often used tool to assess PSC, with a Cronbach’s alpha of 0.88 for the entire question naire and varying between 0.46 and 0.84 for the different dimensions [17].

### Motivation questionnaire

a validated motivation questionnaire based on Maslow and Herzberg theories was employed [18, 19, 20].

## Operational definition of variables

### Hospital survey on patient safety culture (HSOPSC) [21, 16]

The questionnaire used explored ten dimensions of patient safety culture: Overall perception of safety: 4 items. Frequency of reporting adverse events: 3 items. Supervisors’ expectations and actions regarding patient safety: 4 items. Learning organization and continuous improvement: 6 items. Teamwork within the operating room: 4 items. Freedom to speak up: 3 items. On-punitive response to error: 3 items. Human resources: 3 items. Management support for patient safety: 4 items. Teamwork between hospital units: 6 items. Each dimension comprised multiple items. The questionnaire contained 45 items organized into seven sections. Participants responded using a four-point Likert scale. In the context of the HSOPSC, the 4-level Likert scale is used to assess various aspects of patient safety culture. Respondents choose from four response options, which represent different degrees of agreement or disagreement with the statements in the questionnaire. The use of a 4-level scale forces respondents to take a position, as it eliminates the neutral or undecided option [22].

A dimension was considered well-developed if it scored 75% or higher, underdeveloped if it scored 50% or less, and partially developed if it scored between 50% and 75% [16].

### Motivation questionnaire [18, 19, 20]

This 34-item questionnaire explored both intrinsic and extrinsic motivation factors. Two additional items assessed overall motivation level and intention to change workplaces. A four-point Likert scale was used. Intrinsic factors: Job attributes: Measured by 9 items, focusing on job variety, recognition, etc., and achievement: Measured by 3 items, related to job pride, advancement, and professional development. Extrinsic factors: Compensation: Measured by 11 items, including salary, benefits, work conditions, etc. Collaboration: Measured by 9 items, exploring social climate, teamwork, relationships with colleagues and patients, etc.

### Statistical data analysis

Data entry and statistical analysis were performed using SPSS (Statistical Package for the Social Sciences) version 26.0. The Pearson’s chi-squared test or Fisher’s exact test were used to compare frequencies between different categories, depending on the conditions for their application. The Chi-Square test is specifically appropriate for assessing the association or independence between two categorical variables. The significance level was set at 5%.

For negatively worded questions, we recoded the responses. To calculate the percentage of positive responses, we summed the number of responses corresponding to categories indicating a positive perception and divided by the total number of responses for that question, then multiplied by 100.

### Ethical considerations

Ethical and deontological rules were strictly adhered to throughout this study. Administrative authorization was granted by the heads of the various operating rooms. Participation was voluntary, and written consent was obtained. Data anonymity was ensured.

This study was conducted in accordance with the Declaration of Helsinki. Ethical approval was obtained from the Ethics Committee of Sahloul University Hospital, Sousse (approval number: HS 20-2024).

## Results

### Study population

A total of 236 out of 288 participants responded to our study, resulting in a participation rate of 81.94%. Sahloul University Hospital, with a larger general population than Farhat Hached University Hospital, exhibited a high participation rate of 89.28%. Regarding participation rates by grade, senior operating room instrumentation technicians and operating room nurses showed particularly high participation rates, at 100% and 96.55% respectively. As for residents and senior anesthesia technicians, the participation rates were 89.16% and 86.96% respectively.

### Socio-professional data

A total of 236 operating room staff from two Sousse university hospitals participated, including 27.97% residents and 23.72% OR nurses. The majority (72.03%) were women, aged 36–40 on average. Over a quarter had over 11 years’ overall experience, with nearly 40% having worked in their current department for at least 6 years. The majority (63.56%) of our sample worked at Sousse’s Sahloul University Hospital, primarily in general surgery (33.48%) and ophthalmology (16.10%). Nearly all respondents (96.61%) spent more than half their time in the operating room. Moreover, 95.33% had participated in risk management initiatives within the hospital, and 77.11% had received patient safety training (Table 1).

**Table 1:** Socio-demographic characteristics of study participants (*N* = 236)

Frequency		Percentage (%)
Grade:		
Physician	**44**	**18.64**
Resident	**74**	**31.36**
OR nurses	**56**	**23.72**
SOIT	**22**	**9.32**
SAT	**40**	**16.95**
Gender:		
Males	**66**	**27.97**
Females	**170**	**72.03**
Age:		
< 30	**50**	**21.19**
30–35	**57**	**24.15**
36–40	**62**	**26.27**
41–45	**23**	**9.75**
46–50	**34**	**14.41**
> 50 years	**10**	**4.23**
Years of experience:		
Less than 1 year	**14**	**5.93**
1–2 years	**40**	**16.94**
3–5 years	**56**	**23.72**
6–10 year	**60**	**25.42**
11 years and more	**66**	**27.97**
Length of service in the department:		
Less than 1 year	**44**	**18.64**
1–2 years	**50**	**21.87**
3–5 years	**1**	**21.61**
6 years and more	**91**	**38.56**
Workload in the department:		
Lessthan50%	**8**	**3.39**
More than50%	**228**	**96.61**
Completion of patient safety training:		
Yes	**54**	**22.89**
No	**182**	**77.11**
The healthcare institution:		
Sahloul university Hospital	**150**	**63.56**
Hached university hospital	**86**	**36.44**
The operating room :		
General surgery	**40**	**16.95**
Ophthalmology	**38**	**16.10**
Orthopedics	**27**	**11.44**
Max Fac	**22**	**9.32**
Neurosurgery	**24**	**10.17**
ENT	**30**	**12.71**
Emergency	**12**	**5.08**
Burns	**10**	**4.24**
Digestive	**33**	**13.98**
Total	**236**	**100**

### Classification of the 10 dimensions of patient safety culture according to scores

As mentioned in Table 2, the highest score was obtained for dimension D5: Teamwork within departments (58.3%), while the lowest score was recorded for dimension D2: Frequency of reporting adverse events (16.1%). The overall score of the patient safety culture scale is 38.42%.

**Table 2:** Staff perception of safety culture: average positive response rates across 10 dimensions (*N* = 236)

PSC domains	Average positive response (%)
D1: Overall perceptions of safety	**41.53**
Patient safety is never sacrificed to get more work done	**42.38**
Our procedures and systems are good at preventing errors from happening	**24.58**
It is just by chance that more serious mistakes do not happen around here	**38.13**
We have patient safety problems in this facility	**61.02**
D2: Frequency of events reported	**16.1**
When a mistake is made, but is caught and corrected before affecting the patient, it is reported…	**4.23**
When a mistake is made, but has no potential to harm the patient, it is reported…	**14.41**
When a mistake is made that could harm the patient, but does not, it is reported…	**9.67**
D3: Supervisor/Manager expectations and actions promoting patient safety	**46.82**
Manager says a good word when he/she sees a job done according to established patient safety procedures	**22.89**
Manager seriously considers staff suggestions for improving patient safety	**23.72**
Whenever pressure builds up, my manager wants us to work faster, even if it means taking shortcuts	**70.33**
My manager overlooks patient safety problems that happen over and over	**70.33**
D4 : Organizational learning and continuous improvement	**38.98**
We are actively doing things to improve patient safety	**27.54**
Mistakes have led to positive changes here	**28.81**
After we make changes to improve patient safety, we evaluate their effectiveness	**52.12**
We are given feedback about changes put into place based on event reports	**16.94**
We are informed about errors that happen in the facility	**56.78**
In this facility, we discuss ways to prevent errors from happening again	**51.7**
D5: Teamwork within units	**58.3**
People support one another in this facility	**75.84**
When a lot of work needs to be done quickly, we work together as a team to get the work done	**74.16**
In facility, people treat each other with respect	**78.81**
When one area in this unit gets really busy, others help out	**44.91**
If one operating room is overloaded, the others lend a hand.	**17.8**
D6: Communication openness	**28.25**
Staff will freely speak up if they see something that may negatively affect patient care	**23.71**
Staff feel free to question the decisions or actions of those with more authority	**39**
Staff are afraid to ask questions when something does not seem right	**22.03**
D7: Non-punitive response to error	**48.73**
Staff feel like their mistakes are held against them	**22.46**
When an event is reported, it feels like the person is being written up, not the problem	**51.7**
Staff worry that mistakes they make are kept in their personnel file	**72.03**
D8: Staffing	**22.56**
We have enough staff to handle the workload	**13.56**
Staff in this facility work longer hours than is best for patient care	**16.94**
We rely too much on temporary staff to ensure optimal quality of care	**7.2**
We work in ‘crisis mode’ trying to do too much, too quickly	**52.54**
D9: Management support for patient safety	**42.37**
Management provides a work climate that promotes patient safety	**22.03**
The actions of management show that patient safety is a top priority	**42.38**
Management seems interested in patient safety only after an adverse event happens	**64.4**
Units work well together to provide the best care for patients	**40.68**
D10: Teamwork across units	**40.56**
There is good cooperation among units that need to work together	**35.6**
Units do not coordinate well with each other	**33.06**
It is often unpleasant to work with staff from other units	**35.59**
Things ‘fall between the cracks’ when transferring patients from one unit to another	**50**
Important patient care information is often lost during shift changes	**51.7**
Problems often occur in the exchange of information across units	**46.61**
Frequent staff changes are disruptive to patient care	**31.36**

### The number of adverse event reporting forms completed and submitted over the past 12 months

Over the past year, most staff (86.9%) did not submit any adverse event reports (Table 3).

**Table 3:** Distribution of respondents according to the number of adverse events reports you have completed and submitted over the past 12 months (*N* = 236)

Number of adverse event reports	Percentage
No reports	**86.9**
1 to 2	**5.9**
3 to 5	**5.1**
6 to 10	**1.7**
11 to 20	**0.4**
More than 20	**0**

### Level of care safety

Most staff rated care safety as acceptable (61.44%), 32.2% considering it as low, and 4.24% as failing (Table 4).

**Table 4:** Distribution of respondents according to the level of care safety (*N* = 236)

Level of care safety	Average positive response (%)
Excellent	0.43
Very good	1.69
Acceptable	61.44
Low	32.2
Failing	4.24

### Self and activity-related factors (Intrinsic factors)

#### Job attributes factor

Based on the results, the average score for the Job Attributes dimension of intrinsic factors is 2.41 ± 0.83. The highest score is for the item “Social recognition of your work” with 2.98 ± 0.74, and the lowest score is for the item “Participation in decision-making within the department” with 2.08 ± 1.02.

#### Achievement factor

For the achievement dimension of intrinsic factors, the average score is 2.64 ± 0.9. The highest score is 2.93 ± 0.85 for the item “The pride you feel in your work” and the lowest score is 2.49 ± 0.92 for the item “Participation in continuing education and professional development sessions.

### Factors related to the material and social work environment (Extrinsic Factors)

#### Compensation factor

The table below presents the average score for the compensation dimension of extrinsic factors, which is 1.71 ± 0.7. The highest score is 2.08 ± 0.72 for the item “The daily workload you carry” and the lowest score is 1.42 ± 0.62 and 1.42 ± 0.57 respectively for the items “The salary you receive relative to the work you do” and “The allowances and bonuses you receive (on-call allowance, performance bonus).

#### Collaboration factor

For the collaboration dimension of extrinsic factors, the average score is 2.55 ± 0.8. The highest score is 3.11 ± 0.78 for the item “The relationship with patients and their families” and the lowest score is 1.92 ± 0.79 for the item “The welcoming policy and social climate in the department.

#### Ranking of motivation factors based on their average scores

According to the results presented in the table below (Table 5), the ranking of motivation factors in ascending order is: 1.71 ± 0.7 for the “Compensation” dimension, 2.41 ± 0.83 for the “Job Attributes” dimension, 2.55 ± 0.8 for the “Collaboration” dimension, and 2.64 ± 0.9 for the “Achievement” dimension. The average score of factors influencing work motivation is 2.33 ± 0.81.

**Table 5:** Ranking of motivation factors based on their average scores (*N* = 236)

Factor	Average Score	Standard Deviation	Rank
Achievement	2.64	0.9	4
Collaboration	2.55	0.8	3
Job Attributes	2.41	0.83	2
Compensation	1.71	0.7	1
Overall Average	2.33	0.81	

### Motivation level

**Fig. 2:**
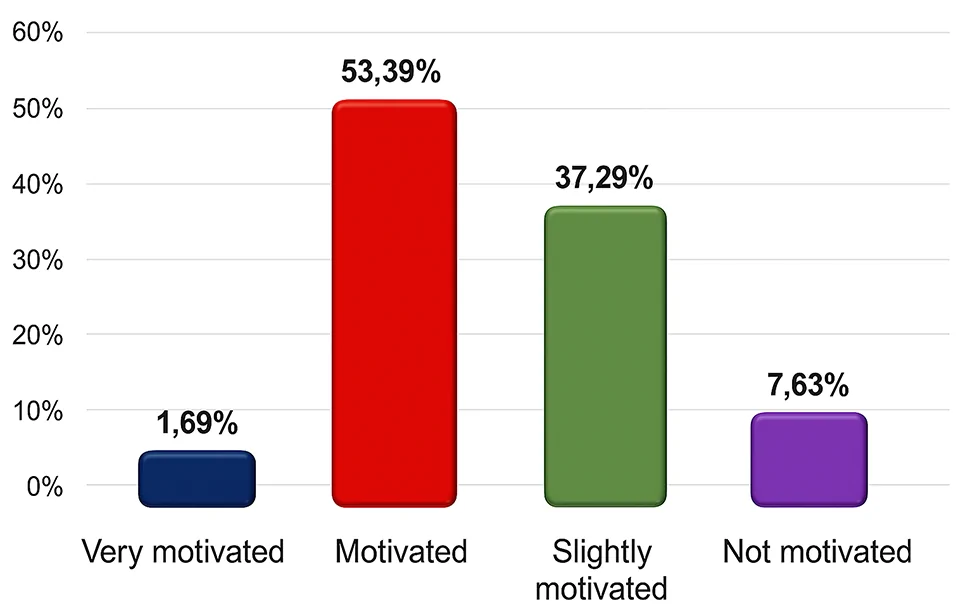
Employee distribution based on self-reported motivation levels

Overall, more than half of the staff, or 53.39%, are motivated in their work (Fig. 2).

### Correlation study

The table below (Table 6) reports a correlation study between the scores of the 10 dimensions of safety culture and the scores of motivation factors. It shows that there is a relationship between dimension D1: Overall perception of safety and compensation (*p* = 0.023), between dimension D2: Frequency of reporting adverse events and achievement (*p* = 0.018), between dimension D3: Expectations and actions of hierarchical superiors regarding patient safety (*p* = 0.032), between dimension D4: Learning organization and continuous improvement and job attributes (*p* = 0.046) and achievement (*p* = 0.022), between dimension D6: Freedom of expression and job attributes (*p* = 0.004), between dimension D8: Human resources and collaboration (*p* = 0.027) and achievement (*p* = 0.002), and dimension D10: Teamwork between departments of the institution and compensation (*p* = 0.005).

**Table 6:** Relationship between the scores of the 10 dimensions of safety culture and the scores of motivation factors based on their average scores

Dimension	Compensation	Job Attributes	Collaboration	Achievement
D1: Overall perception of safety	**0.023**	0.061	0.053	0.241
D2: Frequency of reporting adverse events	0.054	0.163	0.075	**0.018**
D3: Expectations and actions of superiors	0.062	**0.032**	0.083	0.64
D4: Learning organization and continuous improvement	0.325	0.046	0.526	**0.022**
D5: Teamwork within units	0.078	0.360	0.052	0.091
D6: Communication openness	0.054	**0.004**	0.275	0.279
D7: Non-punitive response to error	0.051	0.214	0.073	0.426
D8: Staffing	0.086	0.228	**0.027**	**0.002**
D9: Management support for patient safety	0.556	0.091	0.217	0.063
D10: Teamwork across units	**0.005**	0.051	0.086	0.423

## Discussion

The objective of our study was to assess the level of development of the patient safety culture and the motivation of staff working in the operating rooms of two university hospitals in Sousse.

In total, 236 professionals provided survey feedback with a response rate of 81.94%. The high response rate indicates strong professional interest in PSC, as was the case for another conducted study in 18 Tunisian adult intensive care units with response rate of 82.37 [23].

Respondents were predominantly residents (31.36%). Women were clearly the dominant group (72.03%). Indeed, the feminization of certain professions continues to grow, such as in France, where women represent a very high proportion of the healthcare workforce (75.9%) [24]. The most represented age group was 36–40 years old, with a percentage of 26.27%. Regarding professional seniority, more than a quarter of participants had 11 or more years of experience (27.97%). This length of service may indicate increased responsibility and awareness in patient care due to accumulated experience and skills. However, it should be complemented by continuous training. According to the data collected, less than 23% of the staff had participated in training programs, indicating a nearly non-existent participation rate.

### Culture safety and improvement recommendation

Our study revealed that the dimension D1 “overall perception of safety” had a low score (41.53%), which was calculated as follows: 61.02% of staff responded positively to the statement, “We have patient safety problems in this operating room.” Additionally, 42.38% responded positively to, “Patient safety is never neglected in favor of increased efficiency.” However, 53.39% gave a negative response to, “Our operations and procedures are effective in preventing errors.” The score for positive responses on the Overall Perception of Safety dimension was 41.53%. This result is consistent with a 2017 study conducted in adult oncology units in Saudi Arabia, which showed that overall patient safety perceptions were on the verge of needing improvement (49%) [25]. Similarly, a 2015 study of 26 Iranian university hospitals found that the overall score for the perception of care safety was 60% [26]. In Tunisia, this dimension was poorly developed (52.45%), according to a multicenter study conducted in primary care centers in 2016 [27]. Thus, another Tunisian mixed-methods study concerning the patient safety culture as perceived by operating room professionals showed a percentage of 33.8% [7].

Our results reveal that the dimension of ‘teamwork within units’ had the highest score (58.3%), but it remains an underdeveloped dimension. Also, a Tunisian mixed-methods study about assessing patient safety culture in 15 intensive care units have shown a score of 48,8% [10]. In contrast, the study by Saber Azami-Aghdash et al. conducted in 2015 found that this dimension had an average score (76.4%) [28]. This reflects a worrying situation, given the importance of teamwork in the care setting. Indeed, several studies have identified teamwork as an essential requirement for better quality of care [23] .

In the other hand, the dimension that had the lowest score was ‘adverse event reporting’ (16.1%), which is similar to the result of the Tunisian study conducted in 2017 by M. Mallouli et al. that found a very low score for this dimension (31.4%) [27]. In another Tunisian study, results have shown that 85.9% of the respondents haven’t reported any AEs in the past 12 months [7]. This under-reporting can be explained by the fact that the commission of error is always considered as a lack of skills and rarely seen as a learning opportunity. Insufficient time to report, lack of feedback, fear of blame, and damage to reputations is considered among the causes [23].

Regarding the dimension “non-punitive response to error”, our study showed a low score of 48.73%.

Also, at 2012 in Egypt, a study have shown that non-punitive responses to errors had the lowest score of 19.5%, representing a dimension that requires improvement [29]. It is essential to establish a culture where individuals are supported to identify and report errors without threat of punitive action or blame.

The hierarchical superior, and particularly the relationship they establish with team members, is essential for improving the quality of work. A harmonious relationship between the superior and the team increases employee satisfaction and motivation, leading to more efficient care and better patient safety [26].Our study showed that the dimension “Expectations and actions of hierarchical superiors regarding patient safety” had a score with a high potential for improvement (46.82%).

Also, the study showed that learning organization and continuous improvement had an underdeveloped score (38.98%). Whereas the study conducted in 2016 at Farhat Hached University Hospital reported that this dimension had an average score (67.8%) [27].

Our findings indicate that healthcare professionals felt discouraged from expressing their opinions (28.25%) within a culture of non-punitive responses to errors. This aligns with the study by MST Pierre et al., which found that only 28% of professionals felt comfortable discussing safety concerns with their supervisors. In nearly 90% of cases where staff remained silent, adverse events occurred [30].

Regarding staffing, our study found that this dimension had a very low score (22.56%). Furthermore, this result confirms what was mentioned in a study conducted in 2016 to measure the level of patient safety culture at CHU Farhat Hached Sousse, which showed that 70% of respondents reported a lack of staff. In the United States, 58% reported a lack of staff, while the lowest percentages were found in Belgium (38%) and Lebanon (36.8%) [31–33]. Enhancement of human resources is an integral part of a continuous cycle of improving patient safety and quality of care.

Considering these findings, healthcare professionals require enhanced patient safety and quality care training. This includes: incorporating patient safety culture into education and continuing professional development ( by complementary courses and certificated sessions). Promote and enforce the consistent use of evidence-based checklists and standardized protocols for routine procedures, surgical safety, and medication administration. Encourage and implement brief daily safety huddles at the start of each shift or day. Implement a clear process for providing timely and constructive feedback to those who report incidents. This feedback should include a summary of the investigation findings, the root causes identified, and the actions taken to prevent recurrence, reinforcing the value of reporting. Involve patients and their families in safety initiatives. Educate them on their role in patient safety, encourage them to ask questions, and provide avenues for them to voice concerns or report incidents themselves.

Those who report incidents should be informed about investigation findings and subsequent actions. Effective human resource management is needed. Implementing a robust human resources management strategy is crucial to optimize staffing levels and working hours [10].

### Motivation and improvement recommendation

In our study, 53.39% of the staff was motivated in their work. This is consistent with two studies conducted in Morocco, one at the CHU Ibn Zohr of Marrakech in 2008 and the other conducted in 2012 at the CHU Al FARABI which found respectively a result equal to 32.5% and 25% [12, 34]. However, a Tunisian study conducted at the CHU Farhat Hached in 2014 showed that more than half of the participants (65%) were “not too little motivated” [15]. A descriptive study conducted in 2018 at the surgical emergency department of Ibn-Rochd Hospital in Casablanca showed that nurses generally demonstrated a moderate level of motivation (64%) [35]. “This can be explained by the fact that determinants of professional motivation in Tunisian public hospitals remain largely unexplored, with few national studies dedicated to this topic, leaving hospital managers with limited insights [15].

**For Extrinsic factors**, this study identified remuneration as the top motivating factor for healthcare workers. Moreover, allowances, bonuses, financial aid, and social benefits represent a crucial motivational element for caregivers. Similarly, a study conducted in Japan in 2011 also demonstrated the positive influence of security on employee motivation [36]. The factor of collaboration ranks third in terms of influence on the motivation of respondents. It explores interpersonal relationships at work. A study conducted in Pakistan in 2014 reported that 38.3% of nurses considered collaboration to be a motivating factor in the public sector [37].

**For intrinsic factors**, the job attribute ranks first for the intrinsic motivation of caregivers. In our study, most participants considered that the diversity of tasks positively influenced the degree of motivation (average score equal to 2.5). Also, this result was approved by the study of Dar S, conducted with nurses in Pakistan in 2014 [37] .Achievement ranks last in the motivation of caregivers. This intrinsic factor represents the most influential motivating factor for healthcare professionals in the studies of Kontodimopoulos N and Lambrou P, which were conducted in Greece and Cyprus respectively [38].

In this sense, several strategies can be implemented. A review of paramedical salaries and benefits could alleviate the feelings of demotivation experienced by a significant portion of the study population. Therefore, a recognition and reward program should be implemented. Additionally, a supervisor’s ability to inspire and guide subordinates, involve them in decision-making, ensure fairness, and encourage individual initiatives positively impacts paramedical staff involvement and motivation. Opportunities for training are also motivating, as they enable healthcare professionals to perform previously difficult or poorly mastered tasks and achieve their personal goals of advancement. Recognizing individual potential allows employees to mobilize all their energy to further earn the trust of their superiors. Regular feedback mechanisms and improvements to working conditions (schedules, rest areas, resources) should also be implemented [15].

### Correlation between safety culture and staff motivation

An analysis of the relationship between safety culture (CS) and motivation has demonstrated a statistical correlation between several dimensions of the Hospital Survey on Patient Safety Culture (HSOPSC) and motivational factors. Indeed, our study showed that the dimension “overall perception of safety” was linked to “remuneration” (*p* = 0.023). This is confirmed by the implementation of performance-based payment systems (P4P) in healthcare systems in several countries such as the United Kingdom (Quality and Outcomes Framework (QOF)), France (Payment by Results (P4P), Individual Practice Improvement Contract (CAPI)). These systems aim to improve the quality of care and the efficiency of the healthcare system by rewarding physicians for their performance on quality indicators [39, 40]. In addition, our results reveal a correlation between the dimension “frequency of adverse event reporting” and achievement (*p* = 0.01). This factor is strongly linked to the frequency of adverse event reporting. This is explained by the fact that reporting adverse events is seen as an integral part of a job well done; staff seek to achieve this sense of satisfaction through reporting adverse events. This is consistent with the results of a study published in the Journal of Nursing Management which revealed that nurses who regularly report adverse effects feel more integrated into the quality and safety processes of their institution, which increases their sense of accomplishment [12].

Moreover, the dimension “expectations and actions of hierarchical superiors regarding safety culture” was associated with the factor “job assignment” (*p* = 0.032), which refers to the process by which employees are assigned to specific positions based on their skills and performance. As for the dimension “learning organization and continuous improvement,” it was significantly associated with both “achievement” (*p* = 0.0226) and “job assignment” (*p* = 0.0461). A learning organization is a concept developed by Peter Senge that describes an organization capable of adapting, changing, and innovating continuously through the individual and collective learning of its members [41].

Furthermore, our results showed a correlation between the 8th dimension “staffing " and the two factors “collaboration“(*p*** = 0.0276)**: and “achievement.” (*p*** = 0.00237). By** comparing the results, we found that 83.06% responded negatively to the statement “We have enough staff to handle the workload” (D8), while the two items “Collaboration with colleagues and teamwork in the department” and “Distribution of employees according to the department’s requirements” had average scores of 2.97 and 2.57 respectively. This highlights the important role of collaboration among colleagues in the operating room to effectively manage demands and staff shortages. Indeed, it allows for an equitable distribution of tasks, optimizes available resources, improves communication, and provides essential emotional support.

Human resources management practices play a crucial role in supporting and improving the factor of employee achievement. By offering development opportunities, providing constructive feedback, recognizing achievements, and creating a positive work environment, HR can significantly contribute to employee satisfaction and personal fulfillment. A strategic and holistic approach to HR is therefore essential to foster a sense of achievement and, consequently, increased motivation and performance within the organization.

### Strengths and limitations

Our study is based on two validated questionnaires, which increases the validity of our results. It is one of the few to analyze the relationship between patient safety culture and the work motivation of staff working in operating rooms. Also, this study is considered the first in Tunisia to focus on this relationship. The results of our study can help healthcare managers implement policies and practices aimed at improving both staff motivation and patient safety. In the other hand, it study was conducted in only two hospitals in the Sousse region, so the results may not be generalizable to other contexts or regions. Besides, The survey period was short (one month). Also, a potential limitation of this study is the presence of social desirability bias. Given the sensitive nature of questions related to patient safety culture and motivation within a critical environment like the operating theatre, participants may have been inclined to provide responses they perceived as more socially acceptable or conforming to institutional expectations, rather than their actual perceptions or behaviors. Some healthcare professionals interviewed lacked interest and motivation due to a lack of understanding, negligence, or ignorance of the concepts involved in our work. Finally, there was a lack of availability of the professionals interviewed due to their workload.

## Conclusion

This study, conducted in operating rooms, aimed to understand the relationship between patient safety culture and staff motivation. It revealed a weak safety culture and low staff motivation, with remuneration being the primary motivator. These results suggest a link between these factors.

Theoretically, this study contributes to the existing literature by providing contextualized evidence from the Tunisian healthcare system, specifically within the critical operating theatre environment. While previous research has explored these constructs independently, our work underscores their intertwined nature in a developing country context, enriching the understanding of how organizational culture directly impacts human factors crucial for patient safety. It highlights the need for a more holistic theoretical framework that integrates both motivational drivers and cultural elements when examining healthcare quality and safety.

Practically, our study provides actionable insights for healthcare administrators. The identified weaknesses necessitate urgent interventions. We emphasize the critical importance of fostering a robust safety culture through unwavering leadership commitment, continuous and targeted training programs (especially in non-technical skills like communication and teamwork), promoting open and transparent communication channels, and implementing regular, comprehensive safety audits. Furthermore, the prominence of remuneration as a motivator strongly suggests that revisiting and optimizing compensation structures could be a powerful lever not only for improving staff motivation but also indirectly for enhancing safety adherence. Also, it is imperative that the Ministry of Health in Tunisia develops and implements a national framework for patient safety culture in operating theatres.

Ultimately, these practical recommendations aim to create a safer environment for patients and a more supportive and engaging workplace for healthcare professionals.

## Supplementary Information

Below is the link to the electronic supplementary material.

Supplementary Material 1

## Data Availability

No datasets were generated or analysed during the current study.
