# Supplementary material for: Relationship between patient safety culture and staff motivation in Tunisian operating rooms: a cross-sectional study
Source: BMC Health Serv Res. 2026 Aug 6;26:1526. doi: 10.1186/s12913-025-13995-w (PMC13732921; doi:10.1186/s12913-025-13995-w)
Supplement: Supplementary file 1 [file 12913_2025_13995_MOESM1_ESM.docx]

**Socio-demographic data:**

Check one answer.

1.You are......

Physician ( )	Resident(s) (	) Operating Room Nurse ( )	TSBO ( )	TSAR ( )

2.You are...

Male (	)	Female (	)

3.You have...... years

4. How many years have you been in your current specialty or occupation?

Less than 1 year ( ) 1-2 years (	)	3-5 years ( ) 6-10 years ( ) 11 years or more ( )

5.How long have you been working in this health facility?

Less than 1 year ( ) 1-2 years (	)	3-5 years ( ) 6 years or more ( )

6.How long have you been working in this operating room?

Less than 1 year ( ) 1-2 years (	)	3-5 years ( ) 6 years or more ( )

7.You work in this operating room ...

Less than 50% of your working time (	)	more than 50% of your working time (	)

8.Do you participate or have you participated in risk management structures or committees at this institution:

Yes (	)	No (	)

9.Have you received patient safety training?

10.Identification of the health facility .............

11.Identification of the operating room .......
